# 5-Androstenediol Ameliorates Pleurisy, Septic Shock, and Experimental Autoimmune Encephalomyelitis in Mice

**DOI:** 10.4061/2010/757432

**Published:** 2010-05-18

**Authors:** Ferdinando Nicoletti, Dominick L. Auci, Katia Mangano, Jaime Flores-Riveros, Sonia Villegas, James M. Frincke, Christopher L. Reading, Halina Offner

**Affiliations:** ^1^Department of Biomedical Sciences, School of Medicine, University of Catania, Via Androne 83, 95124 Catania, Italy; ^2^Harbor Biosciences, 4435 Eastgate Mall, Suite 400, San Diego, CA 92121, USA; ^3^Department of Neurology, Oregon Health and Science University, 3710 SW Veterans Hospital Road, Portland, OR 97201, USA

## Abstract

Androstenediol (androst-5-ene-3*β*,17*β*-diol; 5-AED), a natural adrenal steroid, has been shown to suppress experimental autoimmune encephalomyelitis (EAE) in female SJL/J mice. We here report that 5-AED limits inflammation and proinflammatory cytokines including TNF*α* in murine models of carrageenan-induced pleurisy and lippopolysaccaride- (LPS) induced septic shock. 5-AED binds to and transactivates sex steroid receptors with the same general rank order of potency (ER*β* > ER*α* ≫ AR). 5-AED provides benefit in EAE in a dose-dependent fashion, even when treatment is delayed until onset of disease. The minimally effective dose may be as low as 4 mg/kg in mice. However, benefit was not observed when 5-AED was given in soluble formulation, leading to a short half-life and rapid clearance. These observations suggest that treatment with 5-AED limits the production of pro-inflammatory cytokines in these animal models and, ultimately, when formulated and administered properly, may be beneficial for patients with multiple sclerosis and other Th1-driven autoimmune diseases.

## 1. Introduction

Nonglucocorticoid steroids are subjects of intense scientific investigation as perturbations are associated with various diseases including the pathogenesis of autoimmunity [1]. The “gender gap” [2] with respect to incidence and severity of autoimmune disease has been the focus of efforts to uncover new therapies. For example, estrogens [3] and androgens [4, 5] are protective in several autoimmune disease models, including experimental autoimmune encephalomyelitis (EAE), an animal model for multiple sclerosis (MS).

Recent work has dissociated the anti-inflammatory effect from the neuroprotective effect of estrogen treatment in EAE and has shown that its neuroprotective effects do not necessarily depend on anti-inflammatory properties [6]. Specifically, an estrogen receptor (ER)*α* agonist reduced central nervous system inflammation, whereas an ER*β* agonist treatment did not, but instead, was neuroprotective. Preliminary clinical results were encouraging. In a pilot trial, oral estriol treatment of relapsing remitting multiple sclerosis patients caused significant decreases in enhancing lesions on brain magnetic resonance imaging [7]. However, sex steroid therapy involves serious risks. For example, estrogen treatment involves increased risk for breast cancer in women [8]. Because such estrogen-related toxicities are mediated almost exclusively through ER*α*, ER*β* ligand treatment has been suggested as a potentially safer neuroprotective strategy in multiple sclerosis and other neurodegenerative diseases. Alternatively, we have suggested that risks associated with ER*α* could be minimized by the use of safe synthetic derivatives [9] or low-dose estrogen that produces synergistic effects when used in combination with another immunoregulatory therapy [10]. We have also proposed use of related steroids [11] such as androstenediol (androst-5-ene-3*β*,17*β*-diol; 5-AED) that may result in optimal, simultaneous engagement of multiple sex steroid receptors.

Treatment with 5-AED (40 mg/kg) provided significant benefit when given at disease onset in the SJL/J female mouse model of EAE [11]. 5-AED was also anti-inflammatory as we observed an increased survival in a murine model of LPS-induced shock and reduced neutrophil burden in a murine model of carrageenan-induced pleurisy. Finally, a dramatic increase in the Th2 associated response was observed in the popliteal lymph node assay (i.e., B cell/T cell ratio, antigen specific IgM and IgG1, as well as IL-4 production). Here those observations are confirmed and extended. We now report that 5-AED reduced both inflammatory cells and cytokines (TNF*α*  and IL-6) in murine pleurisy and reduced serum levels of TNF*α* as well as improved survival in LPS-induced shock. 5-AED provided dose-dependent benefit in the SJL/J female mouse model of EAE, with a minimally effective dose of between 40 and 4 mg/kg. In contrast to an injection of a suspension formulation, injection of 5-AED in a solution formulation was not effective. 5-AED binds to and transactivates sex steroid receptors with the same general rank order of potency (ER*β* > ER*α* ≫ AR). Taken together with our clinical observations, these studies suggest that treatment with 5-AED may provide safe and efficacious benefits to patients with MS.

## 2. Methods

### 2.1. Drugs

Test compound androst-5-ene-3*β*,17*β*-diol (5-AED) and vehicles (HERF202 or HERF405) were provided by Harbor Biosciences (San Diego, CA). HERF202 was comprised of 30% *β*-cyclodextrin sulfobutyl ether sodium salt (w/v) in water and formed a solution of the test compound. HERF405 comprised of 0.1% carboxymethylcellulose, 2% polysorbate 80 and 0.1% metabisulfite in phosphate-buffered saline pH 7.4 and formed a suspension of the test compound.

### 2.2. Nuclear Receptor Binding Assays

Assessment of binding activity for various nuclear receptors was performed by homogeneous competition assays using the PolarScreen fluorescence polarization system (InVitrogen, Carlsbad, CA). Briefly, serial dilutions of 5-AED or a reference competitor were incubated on 384-well plates for 2 hours at room temperature in the presence of an appropriate fluorescent ligand (Fluormone) and a nuclear receptor of recombinant origin (AR, no. P3018; GR, no. P2816; ER*α*, no. P2614; ER*β*, no. P2615; PR, no. P2895) in a total volume of 30 *μ*L, following the manufacturer's instructions. Fluorescence polarization (FP) in each well was determined with a GENios Pro reader (Tecan, San Jose, CA), and based on the extent of FP suppression detected, IC_50_ competition values were derived using the GraphPad Prism software (GraphPad, San Diego, CA). Most nuclear receptors employed in these assays are full-length recombinant proteins of human origin, with the exception of AR (His/GST-tagged rat AR ligand binding domain) and PR (GST-tagged human PR ligand binding domain).

### 2.3. Nuclear Receptor Transactivation Assays

Transactivation of sex steroid or corticosteroid receptors was assessed in the stable transfectant human cancer cell lines MDA-kb2 (American Type Culture Collection (ATCC) no. CRL-2713) and T47D-kBluc (ATCC no. 2865), which express AR/GR and ER*α*/*β*, respectively [12, 13]. Briefly, cells were plated at 2 × 10^4^ cells/well in 96-well clear bottom white assay microtiter plates (Corning, Lowell, MA) in 100 *μ*L phenol red-free RPMI-1640 medium supplemented with 4 mM **L**-glutamine and 10% charcoal-stripped fetal bovine serum (CHAR-DEX FBS; InVitrogen). Cells were incubated overnight with the various compounds and luciferase activity was then determined in cell lysates. Transactivation activity of the human mineralocorticoid receptor (MR) and peroxisome proliferator-activated receptors (PPAR-*γ* and PPAR-*δ*) used the fluorimetric Gene Blazer *β*-lactamase assay system (InVitrogen) following the manufacturer's instructions. Assays were conducted on 384-well plates using a fluorogenic substrate (CCF4-AM, InVitrogen). Reference compounds (Tocris, Ellisville, MO) used for each receptor were Rosiglitazone and GW1929 for PPAR-*γ* (EC_50_ = 1.3 nM), L165,041 for PPAR-*δ* (EC_50_ = 1.1 nM), and aldosterone for MR (EC_50_ = 0.02 nM).

### 2.4. Carrageenan-Induced Pleurisy

#### 2.4.1. Animals

6-to-8-week-old CD1 male mice (Harlan, San Diego) were used for the carrageenan- (CAR-) induced pleurisy studies. The animals were housed in a controlled environment and provided with standard rodent chow and water. Animals were housed in the Animal Resource Facility at the La Jolla Institute of Molecular Medicine (San Diego, CA) in accordance with institutional guidelines.

#### 2.4.2. Experimental Groups

Mice (4 per group) were treated by subcutaneous (s.c.) injection either with 100 *μ*L of 5-AED suspension (40 mg/kg) or with vehicle alone 24 hours and 1 hour before CAR challenge. Groups of mice were sacrificed at 4-, 5-, and 6-hour time points after CAR challenge. 

#### 2.4.3. Pleurisy

Mice were anaesthetized with isoflurane and the skin was incised at the level of the left sixth intercostal space. The underlying muscle was dissected and saline (0.1 mL) or saline containing 2% *λ*-CAR (0.1 mL; Sigma Chimica, Milan, Italy) was injected into the pleural cavity. The skin incision was closed with a suture and the animals were allowed to recover. At 4–6 hours after the injection of CAR, the animals were euthanized by inhalation of CO_2_. The chest was carefully opened and the pleural cavity rinsed with 1 mL of saline solution containing heparin (5 U/mL) and indomethacin (10 *μ*g/mL). Any exudate (in these studies, less than 200 *μ*L) and washing solution were removed by aspiration. Granuclocytes were suspended in PBS, and enumerated by FACscan with standard software (BD Biosciences, Franklin Lakes, NJ). Levels of TNF*α* and IL-6 in plural rinse were determined by ELISA (Biosource International, Camarillo, CA).

#### 2.4.4. Statistical Analysis

All values in the figures are expressed as mean ± standard deviation. Results were analyzed by two-sided Student's *t*-test. A *P*  value of <.05 was considered significant. 

### 2.5. LPS-Induced Shock

#### 2.5.1. Mice

Six-week-old CD1 mice (Charles River, Calco, Italy) were kept under standard laboratory (nonspecific pathogen free) conditions at the University of Catania, School of Medicine, and were housed in a controlled environment and provided with standard rodent chow and water. Animal care and use was in compliance with all applicable regulations on protection of animals used for experimental and other scientific purposes. 

#### 2.5.2. Induction of Septic Shock and Experimental Treatment

To induce lethal endotoxemia, the mice were injected *i.p.* with 1 mg LPS (Sigma Chimica, Milan, Italy, code L6011, Lot K4096). Groups of female mice (*n* = 10) were treated (s.c) with either 100 *μ*L of 5-AED suspension (40 mg/kg) or with vehicle 24 hours before or 1, 2, 4, 8, or 24 hours after the LPS challenge. In some studies (performed by Harbor Biosciences) male mice (*n* = 4 per group) were used and serum was taken 2 hours after LPS (0.5 mg) challenge, to measure levels of TNF*α* by ELISA.

#### 2.5.3. Statistical Analysis

Statistical analysis was performed by chi-square for cumulative lethality assessed at 72 hours after LPS injection or by Student's *t*-test. All *P*-values of less than  .05 were considered significant. For statistical analysis each group is compared to that of vehicle-treated control.

### 2.6. Experimental Autoimmune Encephalomyelitis (EAE) 

#### 2.6.1. EAE Studies of 5-AED Suspension

5-AED suspension was tested by F.N. at the University of Catania, Catania, Italy, as in our previous studies [14].

**Mice**

Female SJL/J mice (6 to 8 weeks of age; Charles River Italia, Udine, Italy) were maintained in a pathogen-free vivarium at the University of Catania, School of Medicine (Catania, Italy) receiving *ad libitum* sterilized food and water, and were adapted to the ambient environment for at least 7 days before the start of the study. Animal care and use was in compliance with all applicable regulations for the protection of animals used for experimental and other scientific purposes.

**Induction of EAE and Experimental Treatment**

Relapsing and remitting EAE was induced *via* s.c. injection of (PLP)_(139–151)_  as in our previous studies [14]. Disease symptoms were monitored daily and scored in a blinded manner using the following scale: 0: no illness, 1: flaccid tail, 2: moderate paraparesis, 3: severe paraparesis, 4: moribund state, 5: death. Mice were randomized to each experimental group before immunization to induce EAE. Treatment with 100 *μ*L of either 5-AED (0.4, 4.0 or 40 mg/kg) or vehicle was initiated on Day 8 post immunization, when disease onset was first observed, and continued 6 times per week until Day 30 post-immunization. Statistical analysis of cumulative clinical scores was performed by ANOVA analysis for unpaired data. A *P*-value <.05 was considered to be statistically significant. For statistical analysis, the mice that succumbed to EAE were assigned 5 only for the day of death and then were deleted from the experimental group.

#### 2.6.2. EAE Studies of 5-AED Solution

Studies of soluble *5-AED* solution were performed by H.O. at the Oregon Health Science University, Portland OR, as in our previous studies [11].

**Mice**

Pathogen free female SJL/J mice (6–8 weeks old) were used for these studies. The mice were housed in the Animal Resource Facility at the Portland Veterans Affairs Medical Center in accordance with institutional guidelines.

**Induction of EAE and Experimental Treatment**

Relapsing and remitting EAE was induced *via s.c.* injection of (PLP)_(139–151)_  as in our previous studies [11]. Mice were given s.c injections (100 *μ*L) of either *5-AED* solution (40 mg/kg) or vehicle every day after onset of disease (11 days post immunization to EAE induction). The mice were assessed daily for clinical signs of EAE according to the following scale: 0: no signs; 1: limp tail or mild hind limb weakness; 2: moderate hind limb weakness or mild ataxia; 3: moderately severe hind limb weakness; 4: severe hind limb weakness or mild forearm weakness or moderate ataxia; 5: paraplegia with no more than moderate forelimb weakness; 6: paraplegia with severe forelimb weakness or severe ataxia or moribund condition.

## 3. Results

### 3.1. Differential Engagement of Sex Steroid Receptors by 5-AED

5-AED does not transactivate human mineralocorticoid, glucocorticoid, or peroxisome proliferator-activated receptors (PPAR-*γ* and PPAR-*δ*) (data not shown).

5-AED binds competitively to the sex steroid receptors in a specific rank order (ER*β* > ER*α* ≫ AR). It binds with relatively high affinity to both ER*α* and ER*β*, but only modestly to AR, and it is nearly equivalent to 17*β*-estradiol ER*β* binding (10 nM versus 7 nM; see Table 1). Three experimental systems were used to obtain information regarding the capacity of 5-AED to transactivate these sex steroid receptors (Table 2). As observed in binding experiments, the same general rank order of potency was observed for transactivation activity. In contrast to the much higher activity observed in systems expressing any form of ER, 5-AED exhibits poor AR transactivation. Despite its clear ER transactivation properties at low nanomolar levels, 5-AED appears to be a modest transactivator of human estrogen receptor compared to 17*β*-estradiol (see Table 2).

### 3.2. Effect of 5-AED in CAR-Induced Pleurisy

**Figure 1:**
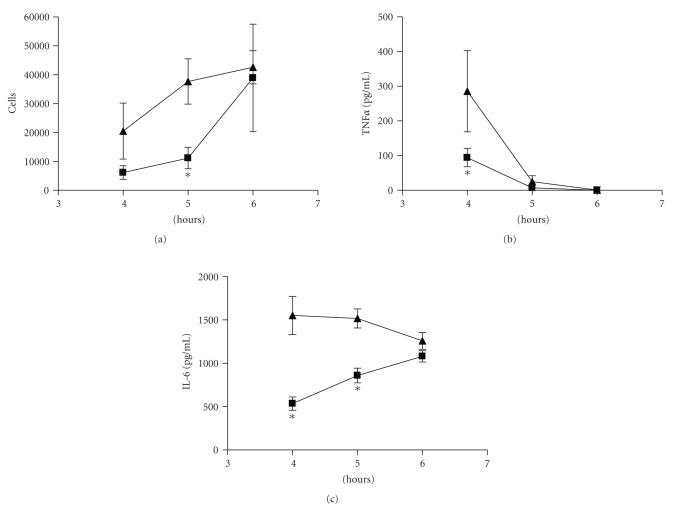
Effect of 5-AED on numbers of PMN and cytokine levels in murine model of CAR-induced pleurisy. Male CD-1 mice (*n* = 4 per group) were anaesthetized with isoflurane, and saline (0.1 mL) or saline containing 2% *λ*-CAR (0.1 mL) was injected into the pleural cavity. Mice were treated (s.c.) with 5-AED in vehicle (squares) or with vehicle alone (triangles) 24 hours and 1 hour before CAR challenge. At 4–6 hours after the injection of CAR, the pleural cavity was rinsed with 1 mL of saline solution. Any exudate (less than 0.2 mL in these studies) and washing solution was removed by aspiration. The leukocytes were suspended in phosphate-buffer saline (PBS) and evaluated by FACscan analysis of live cells in suspension. Cytokine levels were measured by ELISA. All values in the figures expressed as mean ± one standard deviation. These studies were repeated twice with similar results. *Indicates significant (*P* < .05) difference when compared to control values.

All mice, which had received CAR and were treated with vehicle alone, developed an acute pleurisy with increased numbers of infiltrating cells (Figure 1). The peak of the cellular response was between 4 and 6 hours after administration of CAR. Relative to the negative control mice (vehicle alone), treatment with 40 mg/kg 5-AED suspension significantly reduced the degree of lung-associated inflammation as judged by reduced cellular infiltration (at 5 hours) and levels of pro-inflammatory cytokines TNF*α* and IL-6 (at 4 hours and 4 and 5 hours, resp.). By 6 hours, the AED-treated group was nearly identical to the non-AED-treated group for all parameters shown. Similar kinetic studies were repeated twice with similar results.

### 3.3. Effect of 5-AED on Serum Levels of TNF*α* and Survival in LPS-Induced Shock

**Figure 2:**
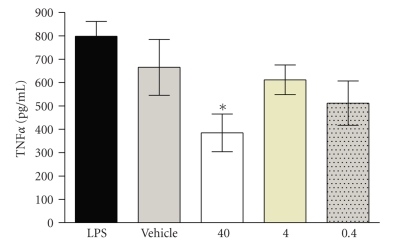
Effect of 5-AED on serum levels of TNF*α* in a murine model of LPS-induced shock. CD-1 mice (males, 4 per group) were treated (s.c. injection) with 5-AED (0.4, 4.0 or 40 mg/kg) 24 hours before and 1 hour after IP challenge with LPS (0.5 mg). 2 hours after the LPS challenge, mice were killed and serum levels of TNF*α* are measured by ELISA. All values in the figures are expressed as mean ± one standard deviation. These studies were repeated three times with similar results. *indicates significant (*P* < .05) difference when compared to control values.

At 1 hour after LPS challenge, serum levels of TNF*α*  were significantly (*P* < .05) reduced in mice treated with 40 mg/kg 5-AED suspension compared to vehicle-treated animals. Lower doses (0.4 and 4.0 mg/kg) were not effective (Figure 2). As reported in our previous studies [11], mice treated with 40 mg/kg 5-AED also experienced improved survival compared to mice treated with vehicle alone (100% lethality), but only if 5-AED was given 24 hours before, or one hour after LPS challenge (50%–60% lethality, resp.). No survival advantage was demonstrated if 5-AED was given 4, 8, or 24 hours after LPS challenge (100% lethality; data not shown). Similar survival studies were repeated three times with similar results.

### 3.4. Effect of 5-AED in EAE

**Figure 3:**
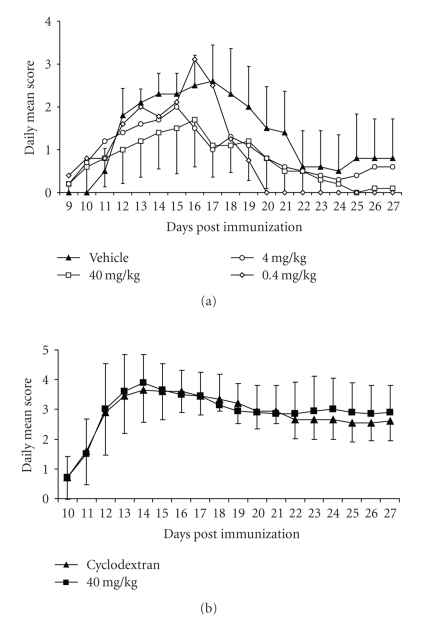
Effect of 5-AED in suspension (a) and soluble formulation (b) on Experimental Autoimmune Encephalomyelitis. Disease was induced in female SJL/J mice (*n* = 10) by subcutaneous immunization with PLP_(139–151)_. Mice began receiving daily s.c. injections with (40, 4 or 0.4 mg/kg) 5-AED (square, open circle, open diamond, resp.) or vehicle (triangle) 8–11 days later (onset). Disease scores were evaluated daily as indicated in Methods. Values in the figure are expressed as mean ± one standard deviation (error bars included for vehicle and 40 mg/kg groups). Statistical analysis of cumulative clinical scores was performed by ANOVA analysis for unpaired data. A *P*  value <.05 was considered to be statistically significant. For statistical analysis, the mice that succumbed to EAE were assigned 5 only for the day of death and then were deleted from the experimental group.

On day 8 post immunization to induce EAE, 5 out of 40 mice (12.5%) showed clinical signs of disease (scores between 1 and 2) and treatment began in all groups. All of these 5 initially effected animals had been randomly assigned to 5-AED groups and none to the vehicle group. First signs of EAE developed in the vehicle group starting on days 10-11. Subsequently, control mice developed a classical course of EAE, with a progressive increase in the clinical score that reached its peak on day 17, followed by recovery (Figure 3(a)). Cumulative disease score was 25.4 ± 9.3. The incidence of EAE was 100% and no lethality occurred in the vehicle group. Incidence was 100% in all groups.

The mice treated with the highest dose of 5-AED exhibited a milder clinical course of the disease entailing a significantly lower cumulative disease score (14.4 ± 9.1) than vehicle-treated control mice. (*P* > .05). A trend toward a milder course of EAE (cumulative disease score 18.1 ± 9.9) was also observed in the mice receiving the 4 mg/kg dose of 5-AED. No lethality occurred in either of these groups. The lowest dose of 5-AED was not effective in view of the unexpectedly high mortality (6/10) after day 17 in this group. Surviving mice in this group experienced a milder course of EAE after day 19 to the end of the study (cumulative disease score 14.5 ± 3.7). The minimally effective dose appeared in the 4 to 40 mg/kg range. In contrast, when 5-AED was given in a soluble formulation (cyclodextran), associated with short half-life and rapid clearance, there were no significant differences between 5-AED- and vehicle-treated groups (Figure 3(b)).

## 4. Discussion

In confirmation of our earlier work [11], we have shown that s.c. injection of 5-AED in suspension ameliorates EAE in female SJL/J mice. The beneficial effect of 5-AED in EAE was dose dependent and may relate to a neuroprotective activity mediated by ER*β* as well as an anti-inflammatory activity, perhaps mediated by ER*α*. Evidence for the later comes from our current observations of reduced or delayed TNF*α* and IL-6 concentrations in exudates and cellular infiltrates in carrageenan-induced pleurisy as well as reduced levels of TNF*α* and improved survival in LPS-induced shock. This confirms our own earlier work [11] and work of others [15, 16] and extends our earlier observations to show that 5-AED treatment only improved 72-hour survival if given 24 hours before or 1 hour after LPS challenge. TNF*α* is a key cytokine implicated in EAE, MS; and many other autoimmune and acute inflammatory diseases [17]. These findings are largely in agreement with earlier work of Suzuki and colleagues [18] and do not discount the potential for involvement of a physiological response to subcutaneous 5-AED particles as a component of the pharmacodynamic in mice. Our data presented here and those of others [19], regarding 5-AED engagement of both ER*α* and ER*β* receptors, suggest that these receptors may be molecular targets consequential to the activity of 5-AED in these models. 

The current studies extend our previous report [11] by showing that the benefit of 5-AED in EAE is dose-dependent. A trend for improvement in cumulative score was observed in the 4 mg/kg group and statistically significant improvement in cumulative score was observed in the 40 mg/kg group. It is unlikely that the unusually high mortality observed only in the 0.4 mg/kg group relates to a drug effect since 5-AED, even at much higher doses, has always been found to be safe and well tolerated in all previous studies, including those in SLJ/J female mice with EAE. Our observation that the 40 mg/kg dose of soluble 5-AED was not effective may relate to the differential drug exposures arising from the different pharmacokinetics of 5-AED when administered in a soluble, quick release formulation versus a suspension form, where the material egresses from the administration site more slowly. When 5-AED (40 mg/kg) was given to mice as a suspension, a C*max* of up to ~100 ng/mL was attained within one hour and significant levels (~10 ng/mL) of 5-AED persisted for as much as 96 hours. These observations are largely in agreement with studies of Singh et al. [20]. In contrast, soluble formulations (cyclodextran) rapidly (within minutes) achieve a high C*max* (<5,000 ng/mL) but with a rapid half-life that results in complete clearance within 8–12 hours, resulting in no drug exposure for half the day (Harbor Biosciences, unpublished observations). However, it remains possible that the failure of soluble 5-AED in the present studies relates to subtle differences in experimental protocols between laboratories and that soluble formulations of 5-AED may provide benefit in EAE under different experimental conditions. 

Published studies demonstrate that benefit in EAE can be achieved by steroidal engagement of either ER*α* or ER*β* receptors, the former is attributed to anti-inflammatory action and the later to neuroprotective effects [6, 21]. The ability of 5-AED to function as a relatively potent ER*β* ligand and only weakly interact with ER*α*  is largely in agreement with earlier work of Blizzard and colleagues [19]. This suggests that benefit of 5-AED in EAE may relate to both properties. The multiple order of the magnitude differences in 5-AED transactivation of either human ER*α* or ER*β* compared to 17*β*-estradiol indicates that receptor activation levels sustained by a 5-AED depot formulation could provide therapeutic benefit to patients with reduced risk of estrogenic side effects. This work has not formally shown involvement of ER*α* or ER*β* in the activity of 5-AED in EAE, nor have we strictly ruled out any role for androgen receptor. It remains possible that other receptors are involved, for example, surface receptors for androgens [22] or estrogens [23]. Although we have shown that 5-AED does not bind to PPAR*γ* or *δ*, the *α* form of the receptor was not tested. This may be particularly interesting in light of the observation that PPAR*α* knockout mice do not respond to DHEA [24].

Extrapolation of an effective dose of 5-AED in patients with MS may find a basis in studies by Tagawa and colleagues [25] when considered along with the pregnancy in multiple sclerosis (PRIMS) study [26]. Tagawa measured serum 5-AED levels before, during, and after pregnancy. Highest levels of 5-AED (6.87 ± 1.95 nM) occur during pregnancy and fall *post partum*, reaching a low of 1.89 ± 0.58 nM within 10 months. The PRIMS study reported on relapse rates in women with MS during the same period. Disease remission in MS is typically observed during pregnancy and increased relapse rates are reported afterwards. Thus, changes in 5-AED in vivo appear to correlate with remitting and relapsing disease, and if so, maintenance of pregnancy levels of 5-AED may reduce relapse rates in patients with MS. This may be achieved with 5-AED sustained release formulations, providing safe therapy to patients with MS and perhaps other Th1-driven autoimmune diseases.

An injectable 5-AED suspension formulation was developed as a countermeasure to exposure to ionizing radiation. This formulation was shown to be safe in Phase I clinical trials and protected primates against ionizing radiation [27]. In our clinical studies, we found that injection of 100–200 mg sustained serum levels of 5-AED (~30 ng/mL) comparable to those seen during pregnancy for several weeks (Stickney et al., in preparation) and sufficient to engage human ER*α* and ER*β* receptors. Taken together with the data reported here and in our clinical studies, we believe that this may be a useful formulation of 5-AED, ready to proceed into phase II testing as treatment for patients with a relapsing remitting form of MS. 

Dehydroepiandrosteone (DHEA), the most abundant adrenal steroid in the body, is the precursor of sex steroids, 5-AED, and other metabolites that have also provided benefit (including improved histology) in the EAE animal model [9]. Attempts are being made to address the metabolic instability and improve the pharmaceutical properties of these low potency natural compounds with synthetic analogues stabilized to metabolism [28, 29]. An injectable suspension formulation, however, may represent an alternative opportunity, where natural compounds, given in depot formulation, release superphysiologic concentrations over a sustained time period. Such an approach may have additional advantages if the downstream metabolites of the endogenous hormone also contribute to the overall therapeutic effect.

The data reported here and in our clinical studies suggest that treatment of humans with an injectable 5-AED suspension formulation (100–200 mg injection) can sustain serum levels of 5-AED comparable to those seen during pregnancy for several weeks, engage ER*α* and ER*β* receptors, and safely achieve both neuroprotection and anti-inflammatory benefits. Thus, our injectable 5-AED suspension formulation may be a useful formulation of 5-AED and is ready to proceed into phase II testing as treatment for patients with a relapsing remitting form of MS. 

## Figures and Tables

**Table 1:** Competition binding IC_50_ values of dihydrotestosterone (DHT), estradiol (E_2_), and androstenediol (5-AED) for AR, ER*α*, and ER*β*.

Ligand	Receptor*		
	AR^(a)^	ER*α* ^(b)^	ER*β* ^(c)^
DHT	15 nM ± 4.2 (4)	ND**	ND
E_2_	ND	8 nM ± 5.4 (3)	7 nM ± 1.6 (3)
5-AED	210 nM ± 44 (4)	49 nM ± 13 (4)	10 nM ± 3.4 (4)

*Results are expressed as IC_50_ values in nM units, representing the statistical mean ± SEM. The numbers in parenthesis represent the number of independent experiments performed (*n* value). See text for details of procedures.

^(a)^AR: Human androgen receptor.

^(b)^ER*α*: Human estrogen receptor *α*.

^(c)^ER*β*: Human estrogen receptor *β*.

**ND: Not determined.

**Table 2:** Transactivation EC_50_ values for dihydrotestosterone (DHT), estradiol (E_2_) and androstenediol (5-AED) for AR, ER*α* and ER*β*.

Ligand	Experimental System*		
	MDA-kb2^(a)^	T47D-kBluc^(b)^	ER*β*-HEK293^(c)^
DHT	0.06 nM ± 0.01 (9)	112 nM (1)	409 nM ± 92 (6)
E_2_	851 nM ± 172 (6)	0.001 nM ± 0.0003 (5)	0.06 nM ± 0.02 (9)
5-AED	2969 nM ± 790 (7)	2.5 nM ± 0.76 (4)	1.7 nM ± 0.26 (7)**

*Results are expressed as EC_50_ values in nM units, representing the statistical mean ± SEM. The numbers in parenthesis represent the number of independent experiments performed (*n* value).

^(a)^MDA-kb2 cells are stably transfected with a promoter/ reporter construct sensitive to sex steroid receptor stimulation fused upstream of (MMTV promoter) a luciferase reporter gene. These cells endogenously express both AR and glucocorticoid receptors.

^(b)^T47D-kBluc cells are stably transfected with a synthetic promoter/ reporter construct sensitive to estrogenic stimulation, consisting of 3 copies of the estrogen response element- (ERE-) fused upstream of a luciferase reporter gene. These cells express endogenously both ER*α* and ER*β*.

^(c)^ER*β*-HEK293 cells are transiently cotransfected with an ERE/luciferase promoter/reporter construct and a cDNA expression vector encoding the full length human ER*β*. These cells exhibit virtually undetectable levels of endogenous sex steroid receptors.

**Although a total of 9 experiments were initially conducted, results from two experiments were discarded as clear outliers on the basis that their values fell >1.5 times the interquartile range above the third quartile of the entire dataset (standard statistical criterion for defining outlier values for a normally distributed population).
